# Administering Docetaxel for Metastatic Hormone-Sensitive Prostate Cancer 1–6 Days Compared to More Than 14 Days after the Start of LHRH Agonist Is Associated with Better Clinical Outcomes Due to Androgen Flare

**DOI:** 10.3390/cancers14040864

**Published:** 2022-02-09

**Authors:** Nicola J. Nasser, Kai Sun, Karen M. Scanlon, Mark V. Mishra, Jason K. Molitoris

**Affiliations:** 1 Department of Radiation Oncology, School of Medicine, University of Maryland, Baltimore, MD 21201, USA; KSun@som.umaryland.edu (K.S.); mmishra@umm.edu (M.V.M.); jmolitoris@som.umaryland.edu (J.K.M.); 2 The Umbilicus Inc., Nonprofit Organization for Preserving Sexual Function of Individuals with Cancer below the Umbilicus, New York, NY 10032, USA; 3 Department of Microbiology and Immunology, School of Medicine, University of Maryland, Baltimore, MD 21201, USA; kscanlon@som.umaryland.edu

**Keywords:** prostate cancer, hormonal therapy, synthetic lethality, combination therapeutics, luteinizing hormone-releasing hormone, testosterone flare, docetaxel

## Abstract

**Simple Summary:**

Hormonal therapy with long-acting luteinizing hormone-releasing hormone (LHRH) for metastatic hormone-sensitive prostate cancer (MHSPC) results initially in testosterone flare followed by testosterone deprivation. Docetaxel is a chemotherapy that is effective against prostate cancer and specifically targets cells during cell division by stabilizing the mitotic spindle, which results in “mitotic catastrophe” and death of the dividing cancer cells. Combining LHRH and docetaxel was proved to be superior to LHRH treatment alone. Here, we show that simply by providing the first dose of docetaxel during testosterone flare, which occurs 1–6 days after LHRH initiation, patients could have better clinical outcomes, as testosterone drives specifically prostate cells into mitosis, priming them to cell kill by docetaxel.

**Abstract:**

Docetaxel, when given at the beginning of androgen deprivation therapy (ADT) for patients with metastatic hormone-sensitive prostate cancer (MHSPC), results in significantly longer overall survival than ADT alone. We aimed to investigate if the delivery of the first dose of docetaxel during the testosterone flare associated with LHRH initiation results in better clinical outcomes, as testosterone induces mitosis of prostate cancer cells, and docetaxel specifically targets cells in mitosis. We analyzed data from the CHAARTED trial which randomized MHSPC patients to ADT alone or ADT plus docetaxel. We included only patients treated with LHRH agonist and docetaxel (*n* = 379). The only cutoff that resulted in differences in treatment outcomes was between patients who started docetaxel 1–6 days (*n* = 18) compared to more than 14 days from LHRH initiation (*n* = 297). Actuarial median overall survival was 72 versus 57 months (*p* = 0.2); progression-free survival was 49 versus 17 months (*p* = 0.06), and freedom from castrate-resistant prostate cancer was 51 versus 18 months (*p* = 0.04) for patients who started docetaxel 1–6 days compared to more than 14 days from LHRH initiation, respectively. Administering docetaxel 1–6 days from the initiation of LHRH agonist for patients with MHSPC could be associated with improved clinical outcomes.

## 1. Introduction

**Figure 1:**
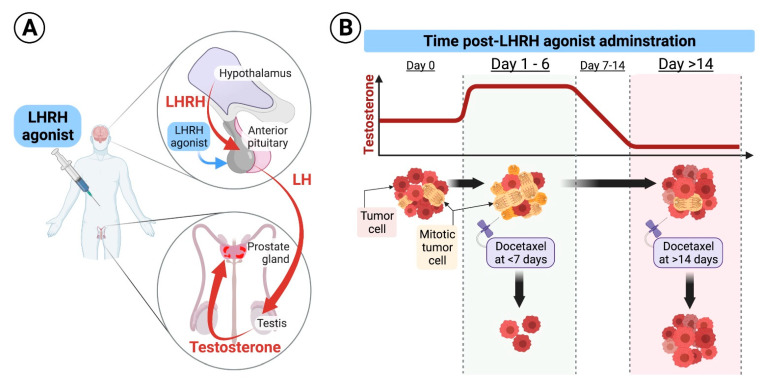
(**A**). Schematic diagram of the LHRH mode of action: Under physiologic conditions, the hypothalamus secretes luteinizing hormone-releasing hormone (LHRH) in a cyclic manner to induce secretion of luteinizing hormone (LH) from the pituitary which results in secretion of testosterone from the testicles. LHRH agonists used for treatment of prostate cancer are formulated in long-acting and continuously released forms that are injected subcutaneously and released to the systemic circulation at high levels for a protracted period of time, ranging from 1–6 months. Continuously released LHRH agonists mask the cyclic secretion of LHRH from the hypothalamus leading to drop in LH secretion from the pituitary and suppression of androgens secretion from the testicles. (**B**). Hypothetical effect of docetaxel at different times of application: Upon initiation of long-acting formulations of LHRH agonists, testosterone levels surges for few days, and then start declining to castrate levels. Testosterone flare results in an increased mitosis of hormone-sensitive prostate cancer cells. We hypothesize that delivery of the first dose of docetaxel during testosterone flare, at days 1–6 after LHRH initiation, results in higher cancer cell kill compared to its delivery when testosterone is at castrate levels, as docetaxel specifically inhibits microtubular depolymerization of the mitotic spindle, which results in cell kill.

Prostate cancer is the most prevalent non-cutaneous malignancy among men [1,2,3]. In 1941, Charles Huggins and Clarence V. Hodges published a series of eight patients with carcinoma of the prostate metastatic to bone who underwent bilateral orchiectomy, with estrogen or androgen injections provided daily before castration [4]. Acid phosphatase activity rapidly decreased following bilateral orchiectomy [4]. Three patients were injected with testosterone, which resulted in an increase in serum acid phosphatase above the pre-injection level [4]. Charles B. Huggins was granted the Nobel Prize in Physiology or Medicine in 1966 for his discoveries concerning hormonal treatment of prostatic cancer [5]. In the 1960s and early 1970s, Roger Guillemin and Andrew Schally discovered the existence of multiple brain peptides controlling hypophysial functions [6,7,8]. The identification of luteinizing hormone-releasing hormone (LHRH) amino acid structure in the early 1970s [7,9] and the development of some of its analogs, long-acting delivery formulations, and antagonists for the treatment of prostate cancer was reported by the group of Dr. Schally and others [7]. Since then, androgen deprivation therapy (ADT) using long-acting LHRH agonists or LHRH antagonists became the backbone of the treatment of metastatic prostate cancer. The Nobel Prize in Physiology or Medicine 1977 was divided, one half jointly to Roger Guillemin and Andrew V. Schally “for their discoveries concerning the peptide hormone production of the brain” [10]. In 2015, ADT plus docetaxel was shown to result in superior survival in the treatment of patients with metastatic hormone-sensitive prostate cancer (MHSPC) compared to ADT alone [11]. GETUG-AFU 15 showed a non-statistically significant difference in survival [12], while STAMPEDE [13,14] and CHAARTED [11,15] trials, which included a larger number of patients, showed a statistically significant survival benefit for adding docetaxel to ADT in the upfront treatment of MHSPC. Fizazi et al. showed that four cycles of docetaxel-based chemotherapy in addition to ADT and local therapy reduces the risk of clinical relapse or death compared to ADT and local therapy alone in men with high-risk localized prostate cancer [16]. The mechanism of synergism between ADT and docetaxel was not addressed in these studies. Androgens have a critical role in driving prostate cancer growth [17]. The use of ADT achieves a drastic reduction in testicular androgen synthesis and in the levels of circulating androgens, thereby suppressing androgen receptor-mediated proliferation of prostate cancer cells [17,18,19]. Docetaxel’s main mechanism of action is the inhibition of microtubule depolymerization in the G2/M phase of the cell cycle, promoting a cascade of events that ultimately leads to cell death [20,21,22]. Other mechanisms of action of docetaxel include the stimulation of apoptosis via down-regulation of the expression of the anti-apoptotic factor Bcl-2 and triggering caspase activation [23,24,25], down-regulation of the ERK1/2 survival signal [26], and inhibition of nuclear translocation of androgen receptor [22,26]. ADT in the CHAARTED trial was achieved by either orchiectomy or a luteinizing hormone-releasing hormone (LHRH) agonist with or without an antiandrogen [11]. We hypothesized that one of the potential mechanisms of synergism between ADT and docetaxel is related to the flare in androgens at the initiation of LHRH therapy [27]. Androgen flare could potentially drive MHSPC cells into mitosis, and thus, if the first course of docetaxel is provided during androgen flare, it could result in higher tumor cell kill and potentially better outcomes (Figure 1). We performed a secondary analysis of the CHAARTED trial [11], in which men with MHSPC were randomized to receive ADT with or without six cycles of docetaxel. We analyzed overall survival (OS), progression-free survival (PFS), clinical PFS, freedom from clinical progression (CP), biochemical control and freedom from castrate-resistant prostate cancer (CRPC) in patients who received LHRH agonist and docetaxel, comparing patients who started docetaxel soon after LHRH initiation, during the expected testosterone flare, to patients who received the first dose of docetaxel when sub-physiologic or castrate levels of testosterone are expected.

## 2. Materials and Methods

### 2.1. Patients

**Figure 2:**
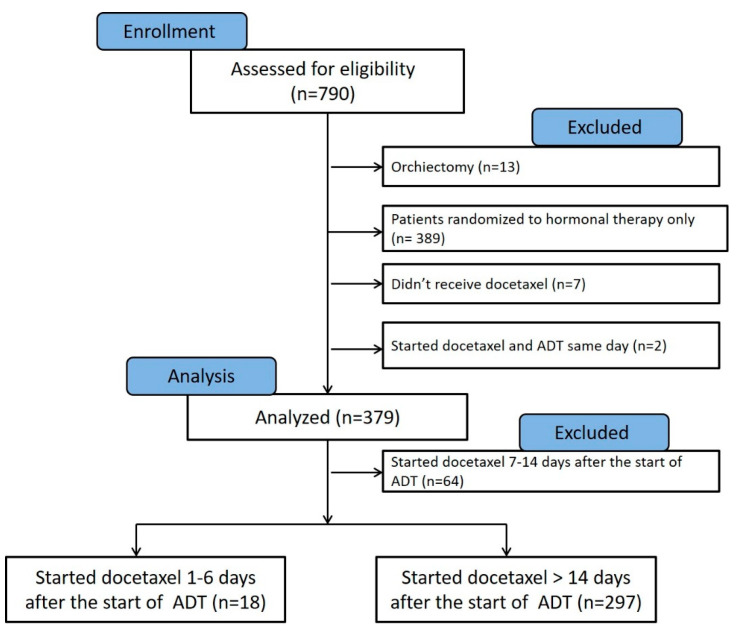
Consort chart showing the patients from the CHAARTED trial that were assessed for eligibility in our analysis (*n* = 790). Patients who underwent orchiectomy (*n* = 13), treated with hormonal therapy alone (*n* = 389), randomized to hormonal therapy and docetaxel but did not receive docetaxel (*n* = 7), and those who started ADT on the same day of receiving docetaxel (*n* = 2) were excluded from analysis. Patients who started docetaxel 7–14 days from the start of ADT (*n* = 64) were excluded from the final Kaplan–Meier, as they likely had varying androgen levels at the time of docetaxel initiation.

This manuscript was prepared using data from Datasets D3, D5, D6, and D7 from the NCTN Data Archive of the National Cancer Institute’s (NCI’s) National Clinical Trials Network (NCTN). Data were originally collected from clinical trial NCT number 00309985, Androgen Ablation Therapy With or Without Chemotherapy in Treating Patients With Metastatic Prostate Cancer (CHAARTED). All analyses and conclusions in this manuscript are the sole responsibility of the authors and do not necessarily reflect the opinions or views of the clinical trial investigators, the NCTN, or the NCI. The full protocol of the CHAARTED trial was previously published as a supplementary document to the original publication [11], and is available at https://www.nejm.org/doi/suppl/10.1056/NEJMoa1503747/suppl_file/nejmoa1503747_protocol.pdf (accessed on 1 February 2022). The analysis of deidentified patients’ data obtained from the NCTN was approved by the Institutional Review Board of the University of Maryland Baltimore, School of Medicine. According to the original study protocol, patients can be randomized to the study within 5 working days before beginning docetaxel. Use of combined androgen blockade was at the investigator’s discretion. Antiandrogens may be used in addition to androgen deprivation therapy but may not be used as the sole hormonal therapy. In the current analysis, we excluded patients who underwent orchiectomy (*n* = 13), randomized to hormonal therapy only (*n* = 389), patients who were randomized to receive docetaxel and did not receive it (*n* = 7), and patients who started LHRH agonist on the same day of docetaxel therapy (*n* = 2) (Figure 2).

### 2.2. Statistical Analysis

Patients were stratified according to the number of days between the initiation of LHRH agonist and docetaxel administration. The Kaplan–Meier method was used to estimate actuarial median OS, PFS, biochemical control, freedom from CRPC, freedom from CP, and clinical PFS. OS is the time interval from randomization to death or date last known alive. PFS is the time interval from randomization to prostate-specific antigen (PSA) progression, clinical progression or death, whichever occurred first, or time from randomization until last known progression-free date. Freedom from CP is the time interval from randomization to clinical progression; patients without documented progression were censored at the date of last disease assessment. Clinical PFS is the time interval from randomization to clinical progression or death, whichever occurred first, or time from randomization until last known progression-free date. CRPC is PSA progression or clinical progression. All data points were collected as of 23 April 2016. The stratified log-rank test was used to assess differences between groups of patients according to time from LHRH initiation to the start of docetaxel. Subgroup analyses were performed using baseline PSA values, Gleason score, and volume of disease. Patients have high volume of disease if they had visceral metastases or ≥4 bone lesions with ≥1 beyond the vertebral bodies and pelvis [11]. 

## 3. Results

The 379 patients included in the current analysis were ordered according to the number of days between the start of LHRH agonist and the initiation of docetaxel. Multiple comparisons between different time cutoffs were performed using the Kaplan–Meier method. 

### 3.1. Comparison between Patients Who Initiated Docetaxel Therapy 1–6 Days versus >14 Days from the Start of LHRH Agonist

**Figure 3:**
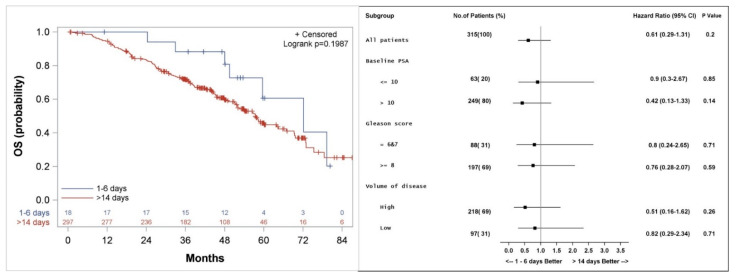
Kaplan–Meier curve (**left**), and subgroup analysis (**right**) for overall survival (OS) for patients who started the first course of docetaxel 1–6 days, compared to more than 14 days after the initiation of LHRH agonist.

**Figure 4:**
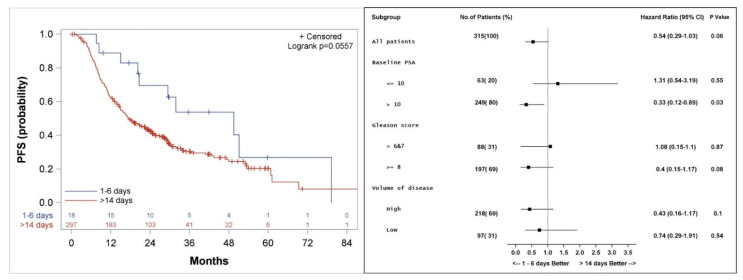
Kaplan–Meier curve (**left**), and subgroup analysis (**right**) for progression-free survival (PFS) for patients who started the first course of docetaxel 1–6 days, compared to more than 14 days after the initiation of LHRH agonist.

**Figure 5:**
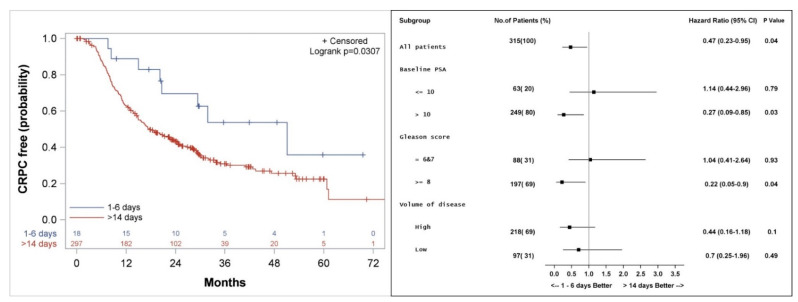
Kaplan–Meier curve (**left**), and subgroup analysis (**right**) for freedom from castrate resistant prostate cancer (CRPC) for patients who started the first course of docetaxel 1–6 days, compared to more than 14 days after the initiation of LHRH agonist.

**Figure 6:**
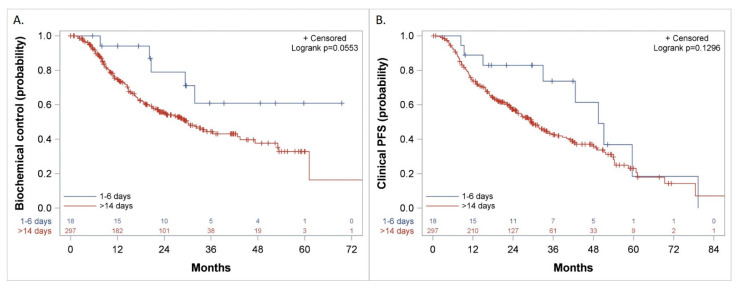
Kaplan–Meier curves comparing patients who started the first course of docetaxel 1–6 days versus more than 14 days after the initiation of LHRH agonist for: (**A**). Biochemical Control, (**B**). Clinical Progression-Free Survival (PFS).

The Kaplan–Meier method was used to estimate differences in actuarial median OS, PFS, clinical PFS, freedom from CP, biochemical control and freedom from CRPC between patients treated with docetaxel closer versus later from the LHRH initiation time point. The only significant differences in clinical outcomes were found between patients who started docetaxel 1–6 days after LHRH initiation (*n* = 18) compared to patients who received the first dose of docetaxel more than 14 days after LHRH initiation (*n* = 297) (Figure 3, Figure 4, Figure 5 and Figure 6). Among patients who received the first dose of docetaxel more than 14 days after LHRH initiation, the median time from LHRH initiation to the start of docetaxel was 46 days (range 15–132 days). Table 1 compares the characteristics of patients who started docetaxel 1–6 days versus >14 days from the initiation of LHRH agonist. There were higher median PSA (60.25 vs. 10.35 ng/mL) and a higher number of patients who did not have local therapy before receiving treatment for metastatic disease (78 vs. 44%), among the patients in the >14-day group compared to the 1–6-day group.

### 3.2. Overall Survival

Median actuarial OS was not significantly different between the groups and was 72 (Confidence interval: 49.5, 94.5) versus 57 (51.9, 64.5) months (*p* = 0.2) for patients who started docetaxel 1–6 days versus >14 days from initiation of LHRH agonist, respectively (Figure 3). Subgroup analysis shows a trend to a better outcome in all subgroups analyzed when docetaxel is started 1–6 days from the initiation of LHRH agonist (Figure 3). 

### 3.3. Progression Free Survival

Median actuarial PFS was 49 (20.2, 79.3) versus 17 (14.9, 22.6) months (*p* = 0.06) for patients who started docetaxel 1–6 days compared to more than 14 days from initiation of LHRH agonist, respectively (Figure 4). Subgroup analysis shows a better PFS in patients with PSA > 10 ng/mL with a hazard ratio (HR) of 0.33 (*p* = 0.03), and a trend to a better PFS among patients with a high volume of disease (HR 0.43, *p* = 0.1) or Gleason score 8–10 (HR 0.4, *p* = 0.08) when treatment with docetaxel was started 1–6 days from the initiation of LHRH agonist, as compared to more than 14 days. 

### 3.4. Freedom from Castrate Resistant Prostate Cancer

Median actuarial freedom from CRPC was 51 (28.8, 73.2) versus 18 (14.9, 22.8) months (*p* = 0.04) for patients who started docetaxel 1–6 days compared to >14 days from LHRH initiation, respectively (Figure 5). Subgroup analysis shows a longer freedom from CRPC among patients with PSA > 10 ng/mL (HR 0.27, *p* = 0.03), patients with Gleason score 8–10 (HR 0.22, *p* = 0.04), and a trend to a better outcome for patients with high volume of disease (HR 0.44, *p* = 0.1) when treatment with docetaxel was started 1–6 days from the initiation of LHRH agonist, as compared to more than 14 days (Figure 5). 

### 3.5. Biochemical Control, Freedom from CP, and Clinical PFS

The duration of biochemical control was not reached versus 30 (CI: 7.6, 50.4) months (*p* = 0.06) (Figure 6A), freedom from CP was 49 (CI: 33, 65) versus 32 (CI: 26.6, 40.9) months (*p* = 0.1) and Clinical PFS was 50 (CI: 33, 79.3) versus 29 (CI: 25, 35) months (*p* = 0.13) (Figure 6B) when treatment with docetaxel was started 1–6 days from the initiation of LHRH agonist, as compared to more than 14 days, respectively. 

## 4. Discussion

Our results show that patients with MHSPC who start the first dose of docetaxel 1–6 days from the initiation of LHRH agonist had longer freedom from CRPC compared to those who start it more than 14 days from LHRH initiation. This is more pronounced among patients with PSA > 10 ng/mL or Gleason score 8–10. LHRH agonists are medications with two opposed effects, an initial surge in testosterone, limited to a few days, followed by suppression in testosterone to castrate levels. This hypothesis, generating retrospective analysis, shows that a testosterone surge during docetaxel therapy could result in better tumor control. 

MHSPC are sensitive by definition to androgen deprivation [28,29,30,31], and thus suppression of testosterone to castrate levels by either orchiectomy or a long-acting LHRH agonist, results in a reduction in tumor cell division and metabolic activity. Combining the cytotoxic chemotherapy docetaxel with hormonal therapy resulted in a better OS in the STAMPEDE [13,14] and CHAARTED [11] trails. The mechanism of this synergism was not previously elucidated. Here, we show that testosterone flair could be beneficial when combined with cytotoxic chemotherapy. Testosterone suppression decreases prostate cancer cell division, and when combined with docetaxel, which specifically targets dividing cells, the results can be at best additive and at worst detrimental. The ongoing NCT03069937 trial, a phase II study of docetaxel before castration with degarelix in men with newly diagnosed metastatic prostate cancer, will provide an answer to if initiating ADT before docetaxel will be inferior to initiating ADT after chemotherapy was started [32]. The study treated patients with metastatic prostate cancer with six cycles of docetaxel every 21 days, and the GnRH receptor antagonist degarelix was started either before cycle one, or at cycle five, for seven injections, 28 days apart. The initial results of the study were presented at the American Society of Clinical Oncology annual meeting in 2021, and it will be very interesting to see long-term follow up [32]. 

The synergistic outcomes of combining LHRH agonist and docetaxel should be analyzed with the antagonistic effects of LHRH agonists on androgen levels in mind. LHRH agonists result in a “tsunami” of testosterone that significantly increases tumor cell mitosis, albeit for a limited number of days. The Hero study shows that testosterone flare occurs after LHRH initiation, and that testosterone levels drop to sub-physiologic levels at 3 weeks, and reaches castrate levels at 5 weeks, from initiation of LHRH agonist [27]. Another report showed that following LHRH initiation, testosterone levels surge starting at about 12 h, reach a peak at day 3, and return to baseline levels at day 7 [33]. 

Adding antiandrogen prior to the start of LHRH agonist was regarded as an “insurance” for castration during testosterone surge in many clinical trials. However, bicalutamide, an antiandrogen, when used as monotherapy for patients with prostate cancer, results in a rise in LH, estradiol, and testosterone levels [34]. Bicalutamide is a nonsteroidal competitive inhibitor of androgen receptor; thus, higher testosterone levels seen after its initiation could be part of the body feedback to offset its effect on androgen receptors. Sleep-related erections were investigated in prostate cancer patients treated with bicalutamide monotherapy, and there were no significant modifications in the number of nocturnal penile tumescence episodes, maximum penile circumference and rigidity time before and after therapy [35]. Prostate-specific antigen [2,3] decreases under treatment with bicalutamide, and thus it is often regarded as a “castrating” medication, despite that most patients maintain erections under antiandrogen monotherapy [36]. A large study that randomized more than 8000 patients with locally advanced prostate cancer to bicalutamide 150 mg plus standard care vs. standard care alone found that impotence was similar in both treatment groups, 9.3% (375/4022) with bicalutamide, and 6.5% (263/4031) with standard care alone [36]. Bicalutamide 150 mg monotherapy was associated with significant advantages compared with castration, in terms of sexual interest and physical capacity, in patients with either M0 and M1 stage prostate cancer [37].

Androgen flare affects specifically prostate cells, as the increase in mitosis that it induces is limited primarily to cells expressing the androgen receptor. Thus, the therapeutic index of docetaxel during androgen flare could be higher than during androgen deprivation for treatment of prostate cancer. The side effect profile of docetaxel during androgen flare and androgen deprivation should theoretically be similar for normal tissues in which the cell cycle is not regulated by androgen. 

Androgen deprivation is pivotal for the control of residual metastatic prostate cancer after the completion of chemotherapy. The PEACE-1 study showed that adding abiraterone to ADT plus docetaxel improves both radiologic PFS and OS in patients with MHSPC [38].

The current study is biased because of its retrospective nature. Due to the retrospective nature of the analysis, the groups were not balanced. Among patients who started docetaxel 1–6 days compared to >14 days after initiation of LHRH agonist, more patients have lower PSA levels, lower volume of disease and more favorable Gleason score. Despite this, our subgroup analysis shows that patients with PSA higher than 10 ng/mL or Gleason score 8–10, seems to do better when docetaxel treatment is started 1–6 days from the initiation of LHRH therapy (Figure 3, Figure 4 and Figure 5). Patients who started docetaxel treatment 7–14 days from LHRH initiation likely had varying androgen levels at the time of docetaxel initiation, with some likely having androgen flare, while others having returned to baseline or achieved sub-physiologic or castrate androgen levels. An additional significant limitation of our study is the small number of patients (*n* = 18) who started docetaxel 1–6 days after the initiation of LHRH agonist. 

Mout et al. [39] studied the efficacy of cabazitaxel in castrated mice and mice receiving testosterone. While cabazitaxel was highly effective in castrated mice, it was ineffective in mice receiving testosterone, as cabazitaxel levels in tumors from castrated mice were about 3.5-fold higher. If this is true for docetaxel in the setting of MHSPC, this would contradict our hypothesis.

Prospective studies and retrospective analysis of other prospective studies such as the STAMPEDE trial [14] are needed to validate our results and the treatment paradigm of combining testosterone surge with mitosis-targeting therapies for treating MHSPC. A potential study design would be randomizing patients to start docetaxel 1–3 days versus 21–40 days after the initiation of LHRH. Another potential study would be comparing the combination of either LHRH or gonadotropin-releasing hormone antagonist, such as Relugolix [19,40] or Degarelix [41,42], with docetaxel. Gonadotropin-releasing hormone antagonists do not result in androgen flare, and thus if our hypothesis is correct, they will be associated with inferior clinical outcomes when combined with docetaxel, as compared to LHRH agonists.

Testosterone at supraphysiologic doses in men with CRPC has been reported to produce promising results [43,44], with trials suggesting a clinical benefit in a subset of patients [45].

The current study suggests a new concept in combining hormonal therapy with cytotoxic chemotherapy, with an emphasis on timing, mechanism of action and synergism. This mechanism of synergism between hormonal therapy and cytotoxic chemotherapy could be tested in other hormone-sensitive malignancies, such as breast cancer [46]. If this concept works, then the timing and the type of chemotherapy courses should be potentially coordinated with the menstrual cycle in premenopausal women with hormone receptor-positive breast cancer. Theoretically, providing docetaxel for metastatic estrogen receptor-positive breast cancer when the ovary is in the follicular phase of the menstrual cycle [36] when estradiol approaches peak levels, likely at 10–14 days from the beginning of the menstrual cycle, could be more potent than providing it during other parts of the cycle. Following the same analogy, providing docetaxel for estrogen receptor-negative, progesterone receptor-positive breast cancer could be more effective if the chemotherapy is delivered during the luteal phase of the ovarian cycle, when progesterone levels reach a peak at about 20–24 days from the beginning of the menstrual cycle [47]. This concept needs to be tested in prospective clinical trials. Caution should be exercised before ovarian resection or orchiectomy when performed as part of the treatment of hormone-sensitive breast and prostate cancers, respectively. 

Our study provides a spark of hope that androgen surge rather than androgen deprivation could be the beneficial aspect of LHRH when combined with docetaxel in men with prostate cancer. The timing of LHRH and docetaxel seems to be crucial for obtaining synergism between the two therapies for MHSPC.

## 5. Conclusions

The timing of administering docetaxel in relation to the start of hormonal therapy with LHRH agonist seems to play a significant role in the treatment of prostate cancer. Administering docetaxel 1–6 days from the initiation of LHRH agonist for patients with MHSPC could be associated with improved clinical outcomes.

## Figures and Tables

**Table 1:** Baseline Characteristics of the Patients.

Characteristic	Gap between LHRH and Docetaxel Intiation 1–6 Days (*n* = 18)	Gap between LHRH and Docetaxel Intiation >14 Days (*n* = 297)	*p*-Value
Age (yrs.) Average	63.9	63.4	0.81
Median	64	64	
Range	47–73	42–83	
Race—no. (%)			0.81
Black	1 (6%)	28 (9%)	
White	16 (88%)	258 (87%)	
Other	0 (0%)	3 (1%)	
Unknown	1 (6%)	8 (3%)	
Volume of metastases—no. (%)			0.07
Low	9 (50%)	85 (29%)	
High	9 (50%)	212 (71%)	
ECOG performance status—no. (%)			0.21
0	14 (78%)	204 (69%)	
1	3 (17%)	89 (30%)	
2	1 (5%)	4 (1%)	
Gleason score			0.11
6	0 (0%)	14 (5%)	
7	8 (44%)	66 (22%)	
8	2 (11%)	61 (21%)	
9	5 (28%)	101 (34%)	
10	0 (0%)	28 (9%)	
Unknown	3 (17%)	27 (9%)	
PSA level at start of ADT—ng/mL			0.0004
Median	10.35	60.25	
Range	0.4–859	0.4–8540	
Prior treatment for prostate cancer—no. (%)			0.004
No local therapy	8 (44%)	232 (78%)	
Primary radiation	3 (17%)	16 (5%)	
Prostatectomy	7 (39%)	49 (17%)	

## Data Availability

Requests for sharing of the data analyzed in this study should be directed to the NCTN Data Archive of the National Cancer Institute.

## References

[1] Siegel R.L., Miller K.D., Fuchs H.E., Jemal A. (2021). Cancer Statistics, 2021. CA A Cancer J. Clin..

[2] Nasser N.J., Klein J., Agbarya A. (2021). Markers of Toxicity and Response to Radiation Therapy in Patients with Prostate Cancer. Adv. Radiat. Oncol..

[3] Nasser N.J., Thoms J., Soosaipillai A., Pintilie M., Wang R., Diamandis E.P., Bristow R.G. (2017). Human tissue Kallikreins: Blood levels and response to radiotherapy in intermediate risk prostate cancer. Radiother. Oncol..

[4] Huggins C., Hodges C.V. (1941). Studies on prostatic cancer. Cancer Res..

[5] The Nobel Prize in Physiology or Medicine 1966. https://www.nobelprize.org/prizes/medicine/1966/huggins/facts/.

[6] Guillemin R. (1978). Peptides in the brain: The new endocrinology of the neuron. Science.

[7] Schally A.V., Block N.L., Rick F.G. (2017). Discovery of LHRH and development of LHRH analogs for prostate cancer treatment. Prostate.

[8] Burgus R., Dunn T.F., Desiderio D., Ward D.N., Vale W., Guillemin R. (1970). Characterization of ovine hypothalamic hypophysiotropic TSH-releasing factor. Nature.

[9] Schally A., Arimura A., Baba Y., Nair R., Matsuo H., Redding T., Debeljuk L., White W. (1971). Isolation and properties of the FSH and LH-releasing hormone. Biochem. Biophys. Res. Commun..

[10] The Nobel Prize in Physiology or Medicine 1977. https://www.nobelprize.org/prizes/medicine/1977/summary/.

[11] Sweeney C.J., Chen Y.-H., Carducci M., Liu G., Jarrard D.F., Eisenberger M., Wong Y.-N., Hahn N., Kohli M., Cooney M.M. (2015). Chemohormonal therapy in metastatic hormone-sensitive prostate cancer. N. Engl. J. Med..

[12] Gravis G., Fizazi K., Joly F., Oudard S., Priou F., Esterni B., Latorzeff I., Delva R., Krakowski I., Laguerre B. (2013). Androgen-deprivation therapy alone or with docetaxel in non-castrate metastatic prostate cancer (GETUG-AFU 15): A randomised, open-label, phase 3 trial. Lancet Oncol..

[13] Clarke N.W., Ali A., Ingleby F.C., Hoyle A., Amos C.L., Attard G., Brawley C.D., Calvert J., Chowdhury S., Cook A. (2019). Addition of docetaxel to hormonal therapy in low- and high-burden metastatic hormone sensitive prostate cancer: Long-term survival results from the STAMPEDE trial. Ann. Oncol..

[14] James N.D., Sydes M.R., Clarke N.W., Mason M.D., Dearnaley D.P., Spears M.R., Ritchie A.W., Parker C.C., Russell J.M., Attard G. (2016). Addition of docetaxel, zoledronic acid, or both to first-line long-term hormone therapy in prostate cancer (STAMPEDE): Survival results from an adaptive, multiarm, multistage, platform randomised controlled trial. Lancet.

[15] Kyriakopoulos C.E., Chen Y.-H., Carducci M.A., Liu G., Jarrard D.F., Hahn N.M., Shevrin D.H., Dreicer R., Hussain M., Eisenberger M. (2018). Chemohormonal Therapy in Metastatic Hormone-Sensitive Prostate Cancer: Long-Term Survival Analysis of the Randomized Phase III E3805 CHAARTED Trial. J. Clin. Oncol. Off. J. Am. Soc. Clin. Oncol..

[16] Fizazi K., Carmel A., Joly F., Delva R., Gravis G., Rolland F., Priou F., Ferrero J.-M., Houede N., Mourey L. (2018). Updated results of GETUG-12, a phase III trial of docetaxel-based chemotherapy in high-risk localized prostate cancer, with a 12-year follow-up. Ann. Oncol..

[17] Harris W.P., Mostaghel E.A., Nelson P.S., Montgomery B. (2009). Androgen deprivation therapy: Progress in understanding mechanisms of resistance and optimizing androgen depletion. Nat. Clin. Pract. Urol..

[18] Miyamoto H., Messing E.M., Chang C. (2004). Androgen deprivation therapy for prostate cancer: Current status and future prospects. Prostate.

[19] Dearnaley D.P., Saltzstein D.R., Sylvester J.E., Karsh L., Mehlhaff B.A., Pieczonka C., Bailen J.L., Shi H., Ye Z., Faessel H.M. (2020). The Oral Gonadotropin-releasing Hormone Receptor Antagonist Relugolix as Neoadjuvant/Adjuvant Androgen Deprivation Therapy to External Beam Radiotherapy in Patients with Localised Intermediate-risk Prostate Cancer: A Randomised, Open-label, Parallel-group Phase 2 Trial. Eur. Urol..

[20] Hernández-Vargas H., Palacios J., Moreno-Bueno G. (2007). Telling cells how to die: Docetaxel therapy in cancer cell lines. Cell Cycle.

[21] Pienta K.J. (2001). Preclinical mechanisms of action of docetaxel and docetaxel combinations in prostate cancer. Semin. Oncol..

[22] Ashrafizadeh M., Mirzaei S., Hashemi F., Zarrabi A., Zabolian A., Saleki H., Sharifzadeh S.O., Soleymani L., Daneshi S., Hushmandi K. (2021). New insight towards development of paclitaxel and docetaxel resistance in cancer cells: EMT as a novel molecular mechanism and therapeutic possibilities. Biomed. Pharmacother..

[23] Mohammadian J., Sabzichi M., Molavi O., Shanehbandi D., Samadi N. (2016). Combined treatment with stattic and docetaxel alters the Bax/Bcl-2 gene expression ratio in human prostate cancer cells. Asian Pac. J. Cancer Prev. APJCP.

[24] Kramer G., Schwarz S., Hägg M., Havelka A.M., Linder S. (2006). Docetaxel induces apoptosis in hormone refractory prostate carcinomas during multiple treatment cycles. Br. J. Cancer.

[25] Fabbri F., Amadori D., Carloni S., Brigliadori G., Tesei A., Ulivi P., Rosetti M., Vannini I., Arienti C., Zoli W. (2008). Mitotic catastrophe and apoptosis induced by docetaxel in hormone-refractory prostate cancer cells. J. Cell. Physiol..

[26] Mang J., Merkle K., Heller M., Schüler J., Tolstov Y., Li J., Hohenfellner M., Duensing S. (2017). Molecular complexity of taxane-induced cytotoxicity in prostate cancer cells. Urol. Oncol. Semin. Orig. Investig..

[27] Shore N.D., Saad F., Cookson M.S., George D.J., Saltzstein D.R., Tutrone R., Akaza H., Bossi A., van Veenhuyzen D.F., Selby B. (2020). Oral Relugolix for Androgen-Deprivation Therapy in Advanced Prostate Cancer. N. Engl. J. Med..

[28] Mori K., Mostafaei H., Sari Motlagh R., Pradere B., Quhal F., Laukhtina E., Schuettfort V.M., Kramer G., Abufaraj M., Karakiewicz P.I. (2021). Systemic therapies for metastatic hormone-sensitive prostate cancer: Network meta-analysis. BJU Int..

[29] Li H., Zhang Y., Li D., Ma X., Xu K., Ding B., Li H., Wang Z., Ouyang W., Long G. (2021). Androgen Receptor Splice Variant 7 Predicts Shorter Response in Patients with Metastatic Hormone-sensitive Prostate Cancer Receiving Androgen Deprivation Therapy. Eur. Urol..

[30] Hofmann M.R., Hussain M., Dehm S.M., Beltran H., Wyatt A.W., Halabi S., Sweeney C., Scher H.I., Ryan C.J., Feng F.Y. (2021). Prostate Cancer Foundation Hormone-Sensitive Prostate Cancer Biomarker Working Group Meeting Summary. Urology.

[31] Cattrini C., España R., Mennitto A., Bersanelli M., Castro E., Olmos D., Lorente D., Gennari A. (2021). Optimal Sequencing and Predictive Biomarkers in Patients with Advanced Prostate Cancer. Cancers.

[32] Gourdin T.S., Lilly M.B., Hussain A., Savage S., Clarke H.S., Sion A.M., Grubb R., Sellman D., Dincman T., Mikoll J. (2021). Preliminary results from a phase II trial of docetaxel before castration with degarelix in men with newly diagnosed metastatic prostate cancer. J. Clin. Oncol..

[33] Thompson I.M. (2001). Flare Associated with LHRH-Agonist Therapy. Rev. Urol..

[34] Verhelst J., Denis L., Van Vliet P., Van Poppel H., Braeckman J., Van Cangh P., Mattelaer J., D’Hulster O., Mahler C. (1994). Endocrine profiles during administration of the new non-steroidal anti-androgen Casodex in prostate cancer. Clin. Endocrinol..

[35] Migliari R., Muscas G., Melis M., Garau M., Sorgia M., Scarpa R.M., Usai E. (1991). Monitoring of erection function in patients with prostatic carcinoma treated with Casodex. Arch. Ital Urol. Nefrol..

[36] McLEOD D.G., Iversen P., See W.A., Morris T., Armstrong J., Wirth M.P., Group C.E.P.C.T. (2006). Bicalutamide 150 mg plus standard care vs standard care alone for early prostate cancer. BJU Int..

[37] Iversen P. (2002). Antiandrogen monotherapy: Indications and results. Urology.

[38] Fizazi K., Galceran J.C., Foulon S., Roubaud G., McDermott R., Fléchon A., Tombal B., Supiot S., Berthold D., Ronchin P. (2021). LBA5 A phase III trial with a 2 × 2 factorial design in men with de novo metastatic castration-sensitive prostate cancer: Overall survival with abiraterone acetate plus prednisone in PEACE-1. Ann. Oncol..

[39] Mout L., de Wit R., Stuurman D., Verhoef E., Mathijssen R., de Ridder C., Lolkema M., van Weerden W. (2018). Testosterone Diminishes Cabazitaxel Efficacy and Intratumoral Accumulation in a Prostate Cancer Xenograft Model. EBioMedicine.

[40] Markham A. (2019). Relugolix: First global approval. Drugs.

[41] Steinberg M. (2009). Degarelix: A gonadotropin-releasing hormone antagonist for the management of prostate cancer. Clin. Ther..

[42] Asakawa J., Iguchi T., Tamada S., Yasuda S., Ninomiya N., Kato M., Yamasaki T., Ohmachi T., Nakatani T. (2018). A change from gonadotropin releasing hormone antagonist to gonadotropin releasing hormone agonist therapy does not affect the oncological outcomes in hormone sensitive prostate cancer. Basic Clin. Androl..

[43] Mohammad O.S., Nyquist M.D., Schweizer M.T., Balk S.P., Corey E., Plymate S., Nelson P.S., Mostaghel E.A. (2017). Supraphysiologic Testosterone Therapy in the Treatment of Prostate Cancer: Models, Mechanisms and Questions. Cancers.

[44] Kumar R., Mendonca J., Owoyemi O., Boyapati K., Thomas N., Kanacharoen S., Coffey M., Topiwala D., Gomes C., Ozbek B. (2021). Supraphysiologic Testosterone Induces Ferroptosis and Activates Immune Pathways through Nucleophagy in Prostate Cancer. Cancer Res..

[45] Nordeen S.K., Su L.-J., Osborne G.A., Hayman P.M., Orlicky D.J., Wessells V.M., van Bokhoven A., Flaig T.W. (2021). Titration of Androgen Signaling: How Basic Studies Have Informed Clinical Trials Using High-Dose Testosterone Therapy in Castrate-Resistant Prostate Cancer. Life.

[46] Turner N.C., Ro J., André F., Loi S., Verma S., Iwata H., Harbeck N., Loibl S., Huang Bartlett C., Zhang K. (2015). Palbociclib in Hormone-Receptor–Positive Advanced Breast Cancer. N. Engl. J. Med..

[47] Barbieri R.L. (2014). The endocrinology of the menstrual cycle. Human Fertility.

